# Supplementary material for: Analyses of Transcriptomics upon IL-1β-Stimulated Mouse Chondrocytes and the Protective Effect of Catalpol through the NOD2/NF-κB/MAPK Signaling Pathway
Source: Molecules. 2023 Feb 7;28(4):1606. doi: 10.3390/molecules28041606 (PMC9962284; doi:10.3390/molecules28041606)
Supplement: Supplementary file 1 [file molecules-28-01606-s001.zip › raw data/KEGG/IL-1b_vs_N/Enrich_plot/IL-1b_vs_N.DEG_KEGG_enriched.pdf]

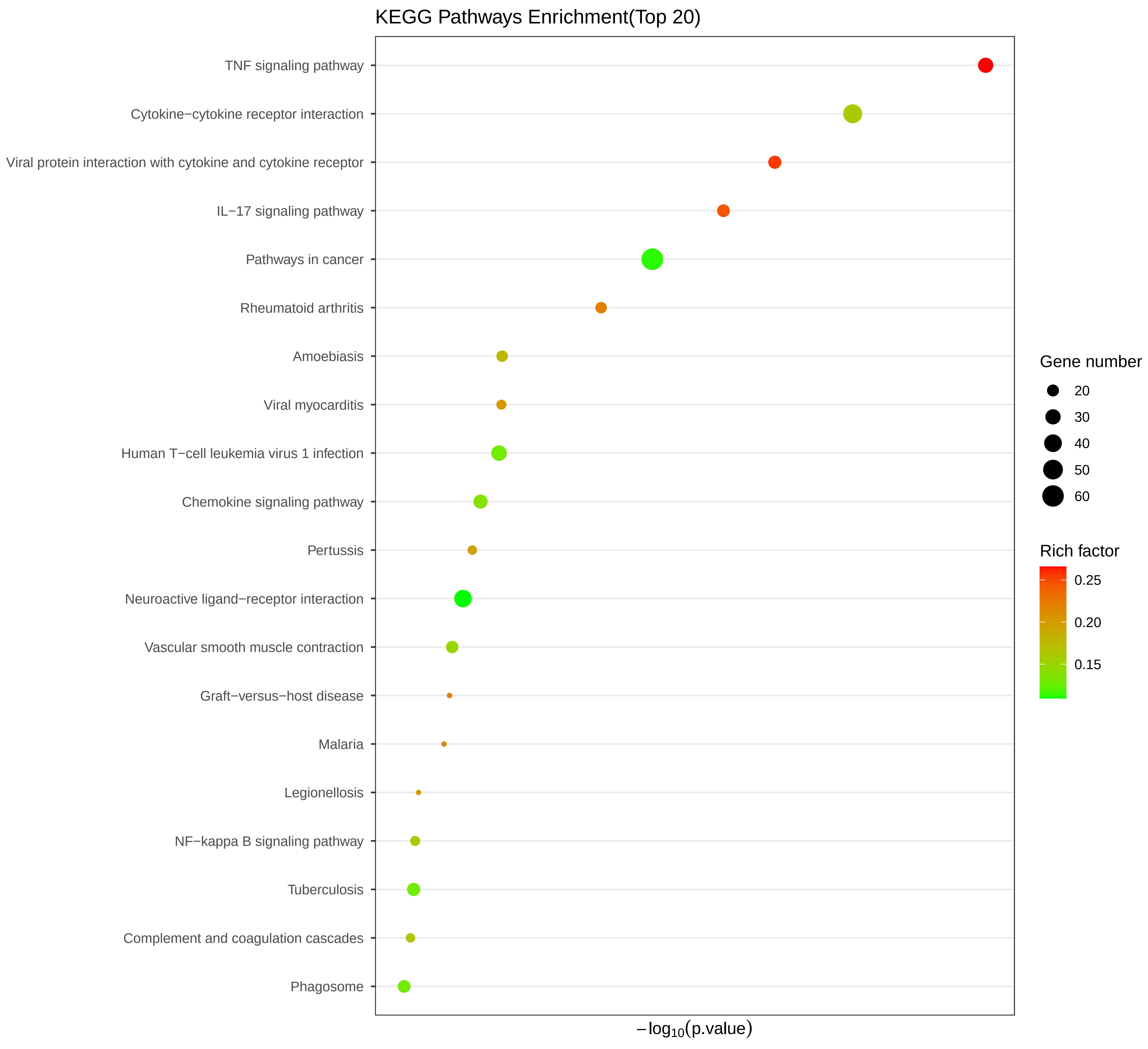
KEGG Pathways Enrichment(Top 20)
TNF signaling pathway
Cytokine−cytokine receptor interaction
Viral protein interaction with cytokine and cytokine receptor
IL−17 signaling pathway
Pathways in cancer
Rheumatoid arthritis
Amoebiasis
Viral myocarditis
Human T−cell leukemia virus 1 infection
Chemokine signaling pathway
Pertussis
Neuroactive ligand−receptor interaction
Vascular smooth muscle contraction
Graft−versus−host disease
Malaria
Legionellosis
NF−kappa B signaling pathway
Tuberculosis
Complement and coagulation cascades
Phagosome
$-\log_{10}$(p.value)
Gene number
20
30
40
50
60
Rich factor
0.25
0.20
0.15
